# Physiological Noise in Cardiorespiratory Time-Varying Interactions

**DOI:** 10.3390/e28010121

**Published:** 2026-01-19

**Authors:** Dushko Lukarski, Dushko Stavrov, Tomislav Stankovski

**Affiliations:** 1 Faculty of Medicine, Ss. Cyril and Methodius University in Skopje, 1000 Skopje, North Macedonia; dushko.lukarski@medf.ukim.edu.mk; 2 University Clinic for Radiotherapy and Oncology, 1000 Skopje, North Macedonia; 3 Faculty of Electrical Engineering and Information Technologies, Ss. Cyril and Methodius University in Skopje, 1000 Skopje, North Macedonia; dushko.stavrov@feit.ukim.edu.mk; 4 Department of Physics, Lancaster University, Lancaster LA1 4YB, UK

**Keywords:** noise, physiological noise, stochastic processes, nonlinear dynamics, coupled oscillators, biological oscillations, cardio-respiratory system, time-varying breathing, Bayesian inference

## Abstract

The systems in nature are rarely isolated and there are different influences that can perturb their states. Dynamic noise in physiological systems can cause fluctuations and changes on different levels, often leading to qualitative transitions. In this study, we explore how to detect and extract the physiological noise, in terms of dynamic noise, from measurements of biological oscillatory systems. Moreover, because the biological systems can often have deterministic time-varying dynamics, we have considered how to detect the dynamic physiological noise while at the same time following the time-variability of the deterministic part. To achieve this, we use dynamical Bayesian inference for modeling stochastic differential equations that describe the phase dynamics of interacting oscillators. We apply this methodological framework on cardio-respiratory signals in which the breathing of the subjects varies in a predefined manner, including free spontaneous, sine, ramped and aperiodic breathing patterns. The statistical results showed significant difference in the physiological noise for the respiration dynamics in relation to different breathing patterns. The effect from the perturbed breathing was not translated through the interactions on the dynamic noise of the cardiac dynamics. The fruitful cardio-respiratory application demonstrated the potential of the methodological framework for applications to other physiological systems more generally.

## 1. Introduction

In nature, systems rarely exist in complete isolation. Almost all natural processes are continuously influenced by internal fluctuations and external perturbations arising from their environment. These influences can originate from interactions with surrounding systems, intrinsic variability within the components themselves, or stochastic fluctuations at microscopic and macroscopic scales [1]. As a result, the dynamics of real-world systems are shaped not only by their internal deterministic laws but also by various sources of noise. Understanding these interactions and their susceptibility to external stochastic influences is therefore essential for interpreting the dynamics of various systems.

This interplay between intrinsic dynamics and external influences is particularly important in biological systems, which are inherently open and adaptive [2]. Living organisms constantly exchange energy, matter and information with their surroundings, and their internal subsystems interact on multiple spatial and temporal scales. These can cause different variability and fluctuations in the dynamics of the system; on one side we have the variability of the deterministic dynamics, which are often treated as non-autonomous dynamics [3,4], and on the other side we have the dynamical noise, which represents all the other random unknown perturbations [5].

In such settings, the dynamic noise in the biological systems is often regarded as physiological noise [6,7,8,9,10]. Even though noise is often taken as something harmful, in biological systems it is not merely a harmful disturbance, but a fundamental component of physiological function. Physiological noise can contribute to flexibility, robustness, and responsiveness in the face of environmental changes [11,12]. For instance, it can cause qualitative transitions in the physiological systems, often pushing them over the tipping point, or, the physiological noise can induce new states, like noise-induced synchronization [13]. Consequently, understanding biological dynamics requires not only the identification of deterministic interactions but also the characterization of stochastic influences that modulate or drive these interactions.

In this study, we explore how to detect and extract the physiological noise from measurements of biological oscillatory systems. In particular, we use dynamical Bayesian inference [14,15,16] to infer stochastic differential equations describing the phase dynamics of interacting oscillators. Here, we infer the deterministic dynamics which can also be time-varying, and importantly here, we also infer the dynamic noise. We will apply the methodological framework on cardio-respiratory signals in which the breathing of subjects varies in a predefined manner [17].

The paper is organized as follows. In Section 2, we lay down the wavelet analysis for observing the variability in the oscillations, after which we present the adaptive dynamical Bayesian inference for extracting the physiological noise, as we also give details for the recordings and the breathing protocols. Next, in Section 3, we present the nature of the respiratory oscillation variability, followed by the main result of the physiological noise for the different subject groups depending on the type of breathing. In the end, we give short discussion in Section 4 about the results.

## 2. Materials and Methods

### 2.1. Wavelet Transform

We first analyzed the time series by using the continuous wavelet transform [18,19,20]. The wavelet transform was used to observe the existence and strength of the cardio-respiratory oscillations and to follow the induced time-variability in the systems. For a given signal x(t), the continuous wavelet transform is given with the equation

(1) $$WT\left(\omega ,t\right)=\int_{0}^{\infty }\psi \left(\omega \left(u-t\right)\right)x\left(u\right)\omega du.$$

where ω denotes the angular frequency, *t* is the time, and

$$\psi \left(u\right)=\frac{1}{2\pi }\left(e^{j2\pi f_{0}u}-e^{\frac{{\left(2\pi f_{0}\right)}^{2}}{2}}\right)e^{-\frac{u^{2}}{2}}$$

is the complex Morlet wavelet, with central frequency $f_{0}=1$, ∫ψ(t)dt=0, and with *i* being the imaginary unit. It is a time–frequency representation containing both the phase and the amplitude dynamics of the oscillatory elements from the analyzed signal and it is used to check whether the subjects’ respiration followed the desired pattern. To extract the time-variability of the main oscillating component in the wavelets, we used the wavelet ridge extraction method [21].

### 2.2. Dynamical Bayesian Inference

Our primary goal in the analysis is to infer and detect the stochastic dynamics from the phase dynamics, subject to noise, which describe the cardio-respiratory interacting dynamics. In order to infer the stochastic dynamics of dynamical interacting systems in terms of oscillations, we used the method of dynamical Bayesian inference [14,15,16,22]. The model for inference is based on the phase reduction approximation [23,24] which can be applied when investigating the cardiac and respiratory systems, as they are both oscillatory and time-varying in nature. When applying the dynamical Bayesian inference method, the time changes in the signals obtained from the oscillatory systems under investigation are measured. The method describes the systems as the solution of a system of stochastic differential equations and infers the parameters of the model that describe the systems and their interactions.

When the interaction between two oscillators is sufficiently week, according to phase reduction theory, their motion can be approximated by their phase dynamics [23,24]. When the phases of the system can be regarded as monotonic change in the variables, the dynamical process can be represented with the system of differential equations:

(2) $$\dot{\varphi_{i}}=\omega_{i}+q_{i,j}\left(\varphi_{i},\varphi_{j}\right)+\xi_{i},$$

where $\varphi_{i}$ is the phase of the i-th oscillator, $\omega_{i}$ is its angular frequency parameter, $q_{i,j}$ is the coupling function describing the influence of the j-th oscillator on the i-th oscillator and $\xi_{i}$ represents the noise. We point out here that $\xi_{i}$ is a *dynamic* noise, which acts inside the differential Equation (2) and can act upon, and introduce changes to, the dynamics. We note that this is different from *measurement* noise ζ, which can act as disturbance directly on the signal, for example as in $\psi_{i}=\varphi_{i}+\zeta$. In the current study we focus on the dynamic noise as physiological noise, while treatment of both, dynamic and measurement noise, can be found in certain earlier publications [25,26] about the same dynamical Bayesian framework. A common assumption for the dynamic noise is that it is white Gaussian noise given by $\xi_{i}\left(t\right)\xi_{j}\left(\tau \right)=\delta \left(t-\tau \right)E_{ij}$, where the symmetric matrix $E_{ij}$ gives the strengths of the noise. In particular, the noise matrix $E_{ij}$ incorporates information about the dynamic noise in each of the two systems, given with components $E_{11}$ and $E_{22}$ in the first and second system, and the correlations between the noises of the different oscillators given by $E_{12}=E_{21}$. These noise components ($E_{11}$, $E_{22}$ and $E_{12}=E_{21}$) are our primary focus in this study, and the main results will express the statistics and deviations of their values for the physiological noise in the cardio-respiratory phase dynamics.

Because the system is periodic in nature, the deterministic dynamics given by the coupling functions [27] can be represented by a Fourier decomposition:

(3) $$q_{i,j}\left(\varphi_{i},\varphi_{j}\right)=\sum_{k=1}^{\infty }\sum_{s=1}^{\infty }c_{i;k,s}e^{i2pk\varphi_{i}}e^{i2ps\varphi_{j}}$$

For a system of two coupled oscillators, reduction to a finite number K of Fourier terms gives the following:

(4) $$\dot{\varphi_{i}}=\sum_{k=-K}^{K}{c_{k}}^{i}\Phi_{i,k}\left(\varphi_{i},\varphi_{j}\right)+\xi_{i}\left(t\right),$$

where $i=\left\{1,2\right\},\Phi_{1,0}=\Phi_{2,0}=1,{c_{0}}^{i}=\omega_{i}$ and the rest $\Phi_{i,k}$ and ${c_{k}}^{i}$ are the K most important Fourier components (in this work we used K=2). With the assumption for white Gaussian noise, the task is then reduced to inference of the unknown parameters of the model:

(5) $$M=\left\{{c_{k}}^{i},E_{ij}\right\},$$

from where the coupling functions $q_{i,j}$ are determined, and with that the underlying interaction mechanisms [27]. We achieve this by utilizing Bayes’ theorem for obtaining the posterior probability density $p_{X}\left(M|X\right)$ of the unknown parameters M given the data X and given the prior probability density $p_{\mathrm{prior}}\left(M\right)$ of the parameters:

(6) $$\begin{array}{c} p_{X}\left(M|X\right)=\frac{\ell \left(X|M\right)p_{\mathrm{prior}}\left(M\right)}{\int \ell \left(X|M\right)p_{\mathrm{prior}}\left(M\right)dM}. \end{array}$$

Here, the likelihood function ℓ(X|M) is obtained through the stochastic integral of the noise term over time, thus leading to the minus log-likelihood function S=−lnℓ(X|M) defined as follows:

(7) $$\begin{array}{cc} S & =\frac{L}{2}\ln|E|+\frac{h}{2}\sum_{l=0}^{L-1}(c_{k}\frac{\partial \Phi_{k}\left(\phi_{.,l}\right)}{\partial \phi }+\\ \; & +{\left[\dot{\phi_{l}}-c_{k}\Phi_{k}\left(\phi {*}_{.,l}\right)\right]}^{T}\left(E^{-1}\right)\left[\dot{\phi_{l}}-c_{k}\Phi_{k}\left(\phi {*}_{.,l}\right)\right], \end{array}$$

where *h* is the sampling time, *L* is the length of the time series, and the summation over *k* is implicit.

We assume that the prior probability of parameters M is a multivariate normal distribution. Then, taking into account the fact that the log-likelihood (7) is of quadratic form, the posterior probability will also be a multivariate normal distribution. The distribution for the parameter vector c, its mean $\bar{c}$, and covariance matrix $\Sigma_{\mathrm{prior}}\equiv \Xi_{\mathrm{prior}}^{-1}$ are then used to recursively obtain the stationary point of *S* using the following:

(8) $$\begin{array}{cc} E & =\frac{h}{L}{\left({\dot{\phi }}_{l}-c_{k}\Phi_{k}\left(\phi_{\cdot ,l}^{*}\right)\right)}^{T}\left({\dot{\phi }}_{l}-c_{k}\Phi_{k}\left(\phi_{\cdot ,l}^{*}\right)\right),\\ c_{k} & ={\left(\Xi_{\mathrm{prior}}^{-1}\right)}_{kw}\;r_{w},\\ r_{w} & ={\left(\Xi_{\mathrm{prior}}\right)}_{kw}\;c_{w}+h\;\Phi_{k}\left(\phi_{\cdot ,l}^{*}\right)\;\left(E^{-1}\right)\;{\dot{\phi }}_{l}+\\ \; & -\frac{h}{2}\frac{\partial \Phi_{k}\left(\phi_{\cdot ,l}\right)}{\partial \phi },\\ \Xi_{kw} & ={\left(\Xi_{\mathrm{prior}}\right)}_{kw}+h\;\Phi_{k}\left(\phi_{\cdot ,l}^{*}\right)\;\left(E^{-1}\right)\;\Phi_{w}\left(\phi_{\cdot ,l}^{*}\right), \end{array}$$

where the summations over l=1,…,L and over the repeated indices *k* and *w* are implicit.

Because the biological systems often can have time-varying deterministic parts, we use a specific information propagation procedure (like a type of Bayes updating) where we assume that the probability density of the parameters is the convolution of two normal multivariate distributions, $\Sigma_{\mathrm{post}}$ and $\Sigma_{\mathrm{diff}}$: $\Sigma_{\mathrm{prior}}^{n+1}=\Sigma_{\mathrm{post}}^{n}+\Sigma_{\mathrm{diff}}^{n}$. The particular form of $\Sigma_{\mathrm{diff}}$ describes which part of the dynamical fields defining the oscillators has changed, and the size of the change. In general ${\left(\Sigma_{\mathrm{diff}}\right)}_{i,j}=\rho_{ij}\sigma_{i}\sigma_{j}$, where $\sigma_{i}$ is the standard deviation of the diffusion of $c_{i}$ in the time window $t_{w}$, and $\rho_{ij}$ is the correlation between the change in the parameters $c_{i}$ and $c_{j}$. We will consider a particular example of $\Sigma_{\mathrm{diff}}$: we assume there is no change in correlation between parameters ($\rho_{ij}=\delta_{ij}$) and that each $\sigma_{i}$ is a known fraction of the relevant parameter, $\sigma_{i}=p_{w}c_{i}$, where $p_{w}$ indicates that the parameter *p* refers to a window of length $t_{w}$. In the adaptive version of this method that we used [22], the values of two parameters of the model are optimized—the time window $t_{w}$ and the propagation parameter $p_{w}$—which lead to an improved inference of the parameters of the model.

### 2.3. Subjects and Breathing Protocols

The data used in this study were collected as part of a previous project designed to determine variations in cardio-respiratory interactions under different breathing patterns [17]. The study included 20 healthy subjects, 13 male (age mean ± std 17–44) and 7 female (age mean ± std 21–33), with no known cardio-respiratory conditions. The investigation was approved by the Ethical Committee of the Medical Faculty at the University Ss. Cyril and Methodius in Skopje, N. Macedonia, and written consent was given by each of the subjects for participation in the study.

**Figure 1:**
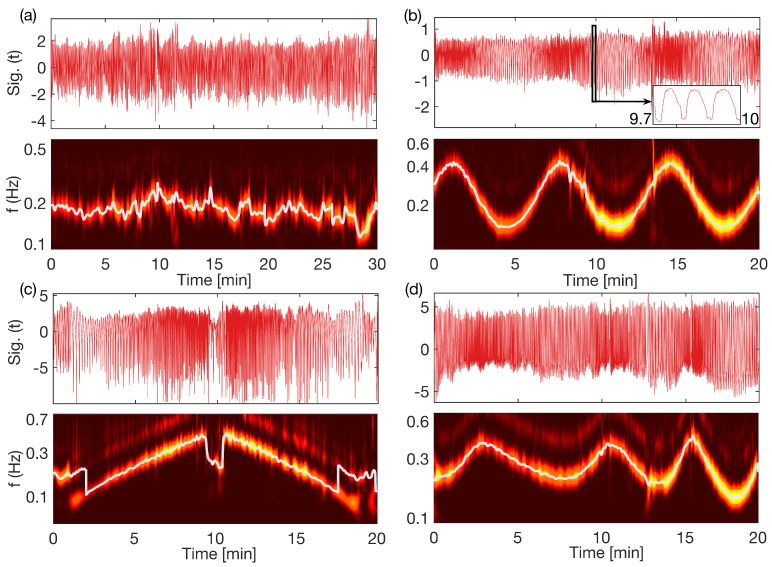
The time and time-frequency wavelet transform of the respiration oscillations during four breathing patterns. The plots in (**a**) represent free spontaneous breathing, in (**b**) sine breathing, in (**c**) linear ramp breathing, and in (**d**) aperiodic breathing of a single subject. On top of every time-frequency plot, with thick white color, we show also the ridge curve.

**Figure 2:**
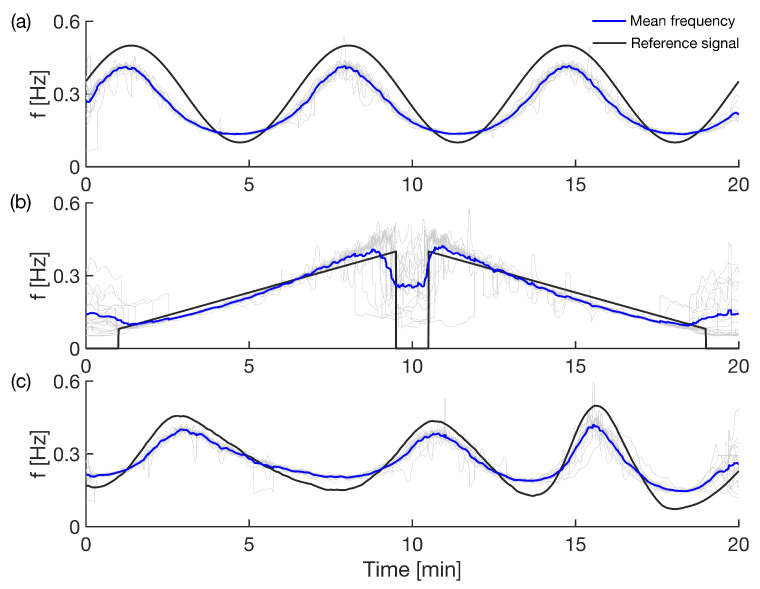
Comparison of the time-varying breathing frequency between the simulated reference and the mean frequency of all subjects. The thin gray lines represent the time-varying frequencies for the individual subjects: (**a**) presents the case for the sine (**b**) the ramp, and (**c**) the aperiodic controlled breathing. The instantaneous time-varying breathing frequency for each subject was extracted by the Wavelet ridge extraction method.

Subjects, lying in supine position, followed a visual and audio computer simulation to adjust their breathing to a predetermined pattern. Figure 1 and Figure 2 show how the subjects followed the specific time-variability during the controlled (a) ramped, (b) sine and (c) aperiodic breathing. Measurements were made using Biotek equipment that obtains respiratory and ECG signals. The respiratory signal is obtained through an electric transducer placed on the subject’s chest that determines the chest circumference, while the ECG signal is obtained with a three-lead ECG measurement. As a limitation, we were not able to measure also the partial pressure of carbon dioxide (${\mathrm{pCO}}_{2}$). The instantaneous cardiac phase was estimated from the ECG signal, while the respiratory phase was estimated from the respiratory signal. Further details of the computer simulation and the measurement setup can be found elsewhere [17].

Measurements were performed for four different breathing patterns: free breathing, ramp-following breathing, periodic sine-pattern breathing, and aperiodic breathing. The duration of the free breathing was 30 min, while the duration of the other three breathing patterns was 20 min each, for each of the subjects. The ramp following breathing consisted of one minute free breathing, followed by linearly increasing breathing frequency for 8.5 min from 0.08 Hz to 0.4 Hz, then 1 min of free breathing, then linearly decreasing breathing frequency for 8.5 min from 0.4 Hz to 0.08 Hz. The periodic sine-pattern breathing followed a sine law change in the frequency, given by the equation f=0.3+0.2sin(2πt/400). The aperiodic breathing pattern followed the z-component of a chaotic Lorenc system [28].

## 3. Results

After acquiring the measurements, the first step in the analysis was to verify that the subjects were breathing according to the predefined patterns. For this purpose, a wavelet transform of the signal was performed for each of the subjects and conformance to the predefined pattern was verified. The time-frequency wavelet transforms for one of the subjects are presented in Figure 1 for different breathing patterns. We can see that the free spontaneous breathing in Figure 1a changes without a specific order, with not so big amplitude variations. The controlled breathing patterns, as shown in Figure 1b–d, followed thr time-variability of the predefined non-autonomous functions of sine, ramp (linear increasing and decreasing) and aperiodic function, respectively. This variability was from one subject; the average breathing frequencies for all subjects, compared to the predefined simulated functions, are shown in Figure 2. The thin gray lines indicate that even though there are some minor deviations for individual subjects, especially for the very high and very low values, the averaged mean frequencies quite closely followed the predefined sine, ramp and aperiodic variations.

After we detected that the frequencies of the respiration have the predefined deterministic time-variability, we then turned to our main focus of detecting the dynamic noise during such time-variability of the frequencies. For this purpose, we infer the whole-phase dynamics of the cardio-respiratory interactions using the adaptive dynamical Bayesian method. This method, in addition to the inference of the parameters that describe the dynamic behavior of the systems and their interactions, also enables the inference of the physiological noise during the investigation of the systems, represented by the noise matrix $E_{ij}$. In particular, we were interested in finding if, and how much, the detected noise differs in relation to the different extents of variations imposed on the breathing frequencies. For this, we analyzed the standard deviations of the inferred noise levels between the subjects in the group during a particular breathing pattern.

**Figure 3:**
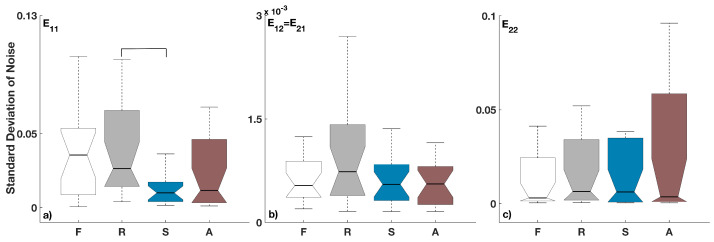
The standard deviation of the noise components $E_{ij}$ for the different breathing patterns: (**a**) $E_{11}$, (**b**) $E_{12}=E_{21}$ and (**c**) $E_{22}$. Statistically significant differences (*p* < 0.05) between different breathing patterns are indicated by bold lines on top of the boxplots. Here, index i=1 stands for respiration and j=2 for the cardiac activity. The four types of breathing are indicated at the bottom as follows: F for free, R for ramped, S for sine and A for aperiodic breathing. For all boxplots, the sample size is constant and equals the number of subjects (*n* = 20).

The standard deviation of the three noise components ($E_{11}$, $E_{12}$ and $E_{12}=E_{21}$) for the different breathing patterns is shown in Figure 3. Statistically significant correlations (*p* < 0.05) between the different breathing patterns are indicated by bold lines on top of the compared boxplots. To control the family-wise error rate, *p*-values were adjusted using the Holm–Bonferroni procedure. The results in Figure 3a show that for the noise in the respiratory dynamics there are significant differences only between the ramp and sine breathing, with the standard deviations for the sine breathing being the lowest. The correlated noise components between the respiration and the cardiac oscillations, as well as the noise components in the cardiac dynamics, had similar levels of deviations and were not significantly different between the different types of breathing—Figure 3b,c.

**Figure 4:**
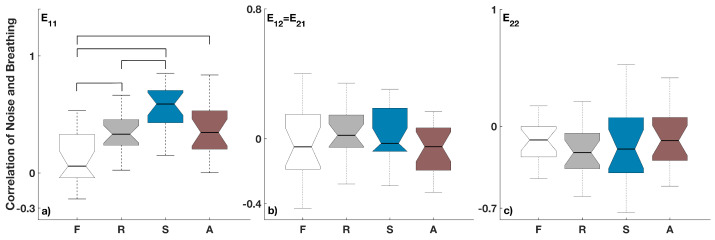
The correlation of noise components $E_{ij}$ to the breathing time variability for the different breathing patterns: (**a**) for $E_{11}$, (**b**) for $E_{12}=E_{21}$ and (**c**) for $E_{22}$. Statistically significant differences (*p* < 0.05) between different breathing patterns are indicated by bold lines on top of the boxplots. The index i=1 stands for respiration and j=2 for the cardiac activity. The four types of breathing are indicated at the bottom as follows: F for free, R for ramped, S for sine and A for aperiodic breathing. For all boxplots, the sample size is constant and equals the number of subjects (*n* = 20).

In addition, we wanted to test whether the detected noise values are changing in accordance with changes in the different breathing patterns. For this reason, we tested the correlation between the inferred noise components and the inferred respiration frequency (${c_{0}}^{1}=\omega_{1}$). Figure 4 shows the correlations between the components of the noise matrix $E_{ij}$ and the time variability of respiration, i.e., the inserted time-varying perturbation to the cardio-respiratory system.

It can be seen that there are statistically significant correlations only for the respiration noise component $E_{11}$ in Figure 4a, while not for the respiration-cardiac noise component $E_{12}=E_{21}$ in Figure 4b or the cardiac component $E_{22}$ in Figure 4c. From the statistical differences of $E_{11}$ in Figure 4a, one can notice that the correlation between the noise and breathing variability is lowest for the free breathing and significantly different from the other three types of breathing. The correlation for the sine breathing, Figure 4a, is the highest, and significantly different both from the free breathing and from the ramp.

## 4. Discussion and Conclusions

Noise is an important part of dynamics of many real systems. Here, we addressed how one can infer the noise, decompose it from the deterministic dynamics and potentially use the information of the noise to assess something for the systems under observation. In this study, we have used dynamical Bayesian inference to infer the stochastic equations of the oscillating model, where the dynamic noise is modeled as white Gaussian noise, as the most general noise with a wide-frequency spectrum. Further generalizations can be performed if another type of noise is used with different types of distributions, for example Poisson (shot) noise or colored Ornstein–Uhlenbeck noise [29].

We were particularly focused on inferring the dynamic noise from biological systems, which is often regarded as physiological noise [6,7,8,9,10]. However, when inferring biological systems, it is important also to account for the possible time-variability and non-autonomicity of the deterministic part. This variability can cause significant changes in the systems and their interaction, leading to qualitative transitions between states of synchronization [30,31,32,33] or oscillation quenching [34,35].

Even though we were primarily focused on the dynamic noise, one should note that the Bayesian method could infer also other disturbances which can affect the precision of the inference. This could happen because the dynamic noise within the method is ultimately calculated as a residual component from the deterministic equation part. Such disturbances could include measurement noise, non-stationarities unrelated to the imposed breathing protocol (e.g., movements, belt tension, etc.), model mismatches caused by the phase reduction procedure, signal preprocessing, or phase detection procedures. In our case, the imposed predefined time-varying breathing protocols were detected with relatively good precision; however, one should always be aware of the above limitations, especially in experimental data, which can affect the precision of the method.

The methodological framework we used is designed for analyzing the interactions and the dynamic noise (Equation (2)). Therefore, even though we focused on the noise, the whole analysis has implications also for the deterministic part and can be used for studying the cardio-respiratory couplings. This is a very active field of research, as noted by a recent review [36], where different methods and approaches have been used for characterizing the cardio-respiratory interactions [9,31,37,38,39,40]. Many such studies have also used the time-varying breathing frequency as a standard perturbation for studying the cardio-respiratory coupling [9,10,15,17,41]. Thus, effective inference and decomposition of the physiological noise indirectly can also lead to a more effective detection of the cardio-respiratory interactions.

The use of the dynamical Bayesian method for the inference of physiological noise had a good effect. However, we point out that other methods can also be used to infer the dynamic physiological noise, which have slightly different performances for different aspects of the interacting dynamics. An important group of such methods are based on transfer entropy and Granger causality [9,10,42] which make explicit use of metrics computed over the physiological noise because they are based on the evaluation of predictability improvement, or the information decrement, in the target dynamics when those of the driver were explicitly accounted for.

In order to test the reliability of our method on time-varying biological interacting systems, we analyzed the cardio-respiratory oscillatory interactions subject to time-varying respiration. Here, we used highly controlled breathing frequency patterns (as ramp, sine and aperiodic functions), which perturbed the cardio-respiratory system by introducing predefined non-autonomous variations in the deterministic part of the dynamics [3,4,43]. These time-varying patterns, as perturbations to breathing frequencies, can be clearly observed in Figure 1 and Figure 2, using the wavelet transform and the wavelet ridge extraction [18,19,20,21].

Because real biological systems often have both time-variability of the deterministic part and dynamic noise, both of which are time-dependent components in the dynamics, we wanted to test how the dynamic noise will be inferred when we inserted explicit time-variability. Translated to our studied cardio-respiratory oscillatory system, this meant that we perturbed the breathing frequency and wanted to see if the inferred dynamic physiological noise will be related to the type of respiration perturbations, and if and how this will be translated through the interactions with the cardiac dynamics. The results in Figure 3a and Figure 4a for the noise in the respiration dynamics showed that there are various statistical differences. These point out that there are mechanisms where the time-varying perturbations of respiration are related and affect the level of the dynamic noise in respiration.

On the other hand, we also investigated whether the respiration perturbations translate influence through the interactions and coupling functions on the cardiac dynamic noise. The results in Figure 3c and Figure 4c for the noise in cardiac dynamics showed that there are no statistical differences compared to free spontaneous breathing or between any of the induced time-varying breathing patterns. Similarly, the joint noise ($E_{12}=E_{21}$) component also showed no statistically significant differences across different breathing patterns.

The application of the methodological framework for the inference of physiological noise from the cardio-respiratory system was very fruitful; however, the method is more general and can be used also with other physiological (oscillatory) systems. For example, in this manner one can infer the physiological noise also from the cardio-respiratory system through measurements of blood flow, airflow breathing or plethysmographic pulse recording [40,44,45]; alternatively, one can apply the noise inference to brain dynamics from EEG and fNIRS recordings [46,47].

## Data Availability

The research data are available upon request to the corresponding author.
